# Do personal and behavioural characteristics of physiotherapy students predict performance during training and course completion?

**DOI:** 10.1186/s12909-023-04070-1

**Published:** 2023-02-07

**Authors:** Sophie Paynter, Ross Iles, Wayne C. Hodgson, Margaret Hay

**Affiliations:** 1 Department of Physiotherapy, Faculty of Medicine, Nursing and Health Sciences, Monash University, Peninsula Campus, 47-49 Moorooduc Highway, Frankston, VIC 3199 Australia; 2 Healthy Working Lives Research Group, School of Public Health and Preventive Medicine, Monash University, Melbourne, VIC 3004 Australia; 3 Faculty of Medicine, Nursing and Health Sciences, Monash University, Clayton, VIC 3800 Australia

**Keywords:** Personality, Approaches to learning, Coping strategies, Academic performance, Clinical competence, Health professional education, Physiotherapy education

## Abstract

**Background:**

Specific personal and behavioural characteristics are required for competent health care practice. Research investigating relationships between these characteristics and course performance of health professions students is expanding, yet little research is conducted within the undergraduate physiotherapy student population. This study aimed to explore the relationships between personality, approaches to learning, and coping strategies of undergraduate physiotherapy students and their performance in academic, clinical and in-course assessment tasks and course progression.

**Methods:**

Participants from six cohorts of undergraduate physiotherapy students (commencing years 2012–2017, 66% response rate) completed questionnaires measuring personality (NEO-FFI-3), approaches to learning (RASI) and coping strategies (Brief COPE). Correlation and multiple regression analysis were conducted to investigate relationships between scores on written examinations, in-course assessment tasks and assessments of clinical performance. Mann–Whitney U test was used to compare subgroups on these measures in those who completed or did not complete the course.

**Results:**

Conscientiousness and a strategic approach to learning predicted higher scores in written examinations, and for most clinical and in-course assessments with conscientiousness being a stronger predictor. A lack of purpose (surface) learning approach was predictive of lower clinical placement scores. Non-course completers had higher scores for lack of purpose (surface) approach to learning and lower scores for the coping strategies of support seeking and humour.

**Conclusions:**

This study confirms the importance of conscientiousness and a strategic learning approach on the academic and clinical performance of undergraduate physiotherapy students. Identifying learners with a surface learning approach and low support seeking coping strategies could assist in providing support to students at risk of poor performance and minimising attrition.

## Background

Quality health care provision requires competent professionals with an increasingly broad array of knowledge, skills and attributes. Irrespective of entry requirements, gaining course entry to health professional degrees is largely restricted to those with high prior academic performance, yet there is variation in performance and attrition during training. The high cost of educating health professionals [1] and the challenges of ensuring the health workforce capacity to meet current and future demands confirm the need for a deeper understanding of factors that influence the performance and retention of health professional students.

Personal attributes necessary for competent clinical practice are listed explicitly throughout competency documents of health professional registration bodies. Competency standards for physiotherapy practice in Australia and New Zealand [2], require that a competent clinician will possess the required knowledge and be trustworthy and conscientious, deliver empathetic and client-centred care, engage in reflective practice to support self-directed and self-regulated learning, and manage their stress [2]. Personal characteristics of health professional students have been an increasing focus of research, primarily in nursing and medicine [3]. Significant relationships have been reported between students’ grade point averages (GPAs) and personality domains [4, 5], learning approaches [6], motivation and self-regulatory factors [7] and perceived stress [8]. Specific clinical outcomes in health professions education are less commonly considered, however, personal attributes assessed during course selection were predictors of the clinical performance of medical [9] and physiotherapy students [10]. Additionally, different behaviour styles have been described in physiotherapy students who received higher and lower scores on clinical placements [11]. Furthermore, resilience and stress reducing activities [12] and personality domains [4, 5] have been correlated with course progression, confirming the importance of investigating the influence of these factors on student performance.

Conscientiousness is the most frequently examined domain from the five-factor model of personality [13]. This model also includes extraversion, neuroticism (low emotional stability), openness to experience and agreeableness. In particular, conscientiousness is considered a determinant of academic performance in tertiary students [3, 7, 14]. The relationship between conscientiousness and clinical performance, primarily reported in medical students, is less consistent [8, 15, 16]. Extraversion has been negatively associated with academic performance [4] but positively related to clinical skill acquisition, where interpersonal style is advantageous [4, 8, 17]. Increased stress, burnout and poor clinical performance have been reported in medical students with high ratings of neuroticism combined with lower conscientiousness and extraversion [4, 17, 18]. Also, clients with chronic disease reported poorer outcomes when managed by physiotherapists who were higher in neuroticism than therapists lower in neuroticism [19]. These aspects have not been investigated in physiotherapy students specifically, where differences may be evident.

Students’ approaches to learning are reported to mediate the effect of certain personality domains on academic performance, specifically, conscientiousness via a strategic approach and openness to experience via a deep approach [20–22]. A learner applying a deep approach seeks understanding, thinks critically, and connects new and established knowledge. In contrast, a learner with a surface approach has little intrinsic interest in the content and is motivated by a fear of failing assessments. A strategic learning approach includes a combination of deep and surface strategies, where the learner is motivated to maximise an assessment's grade rather than achieve mastery of knowledge or memorisation [23]. Deep and strategic learning approaches are prevalent among health professional students, where they typically show positive relationships with academic and clinical assessments, in contrast to surface approaches [14, 22, 24, 25]. A deep approach to learning in medical students has predicted the expression of empathy [26] and students’ future approaches to work as graduates [27]. Deep and strategic approaches to learning are dominant in post-graduate physiotherapy students [28, 29], where strategic approaches have positively correlated with grade point averages [29]. However, relationships between approaches to learning and clinical performance in undergraduate physiotherapy students have not been reported.

Stress is a frequently explored factor impacting performance and course progression of health professions students where higher perceived stress has an adverse effect [3]. Physiotherapy students report stress arising from academic, financial and personal sources, and higher stress levels when undertaking clinical placements [30, 31]. An individual’s response to stress (defined as ‘coping’) may be influenced by personality [32] or approaches to learning [33]. The impacts of stress on individuals vary depending on their coping strategies, which may be positive (i.e. adaptive), resulting in lower feelings of stress relative to maladaptive (i.e. avoidant) strategies, characterised by delays or bypasses in dealing with a stressor [34]. Avoidant strategies have been associated with adverse well-being [35], burnout [36] and poor academic performance [37] of health professional students. Active coping strategies, however, predict better clinical examination performance in early year medical students [38]. The challenges of adapting to new learning environments in preclinical and clinical settings and developing skills to become a competent novice health professional demand much more than acquiring knowledge [30, 39]. Establishing if significant relationships exist between personal characteristics and the performance of physiotherapy students could inform the refinement of course selection processes and tailored student support within courses, both of which may enhance successful course completion.

This study aimed to explore the relationships between personality, approaches to learning, and coping strategies of undergraduate physiotherapy students and their performance on written examinations, in-course assessment, clinical assessment tasks, and course progression. The specific research question was: Do personality, approaches to learning and coping strategies predict physiotherapy students' course performance and course completion? Related hypotheses were 1) conscientiousness and a deep learning approach are associated with higher scores on academic, clinical and in-course assessments and 2) adaptive coping strategies are associated with higher scores in clinical placements.

## Methods

### Setting and participants

Participants from six commencing cohorts (from years 2012 – 2017) of a Bachelor of Physiotherapy degree at a large Australian university were recruited. The degree is a four-year entry to practice qualification comprising 2.5 years of on-campus preclinical education followed by 1.5 years of education in the clinical setting. On-campus units are 12 weeks long, integrating case-based learning of foundational knowledge framed by realistic clinical encounters and skills required for physiotherapy practice. Assessments include those specific to academic knowledge (e.g. written examinations), in-course assessments (e.g. assignments, portfolios and presentations) and clinical competence (Objective Structured Clinical Examinations (OSCEs), and clinical placements).

A pragmatic design was adopted to maximise the participants recruited during the data collection period. This involved varying data collection points relative to the course progression of each cohort. Cohorts 1 and 2 participated in Year 4 prior to graduating, Cohorts 3 and 4 participated as they transitioned to clinical education (in Year 3), and Cohorts 5 and 6 participated in Year 1 of their course. A response rate of 66% was achieved, with 365 participants across the six cohorts providing initial consent and returning at least one completed questionnaire (described below). Individual cohort response rates ranged from 38% (Cohort 6) to 93% (Cohort 3). Participants included 247 (68%) females and 118 (32%) males. Participants comprised local students (from Australia or New Zealand) who had entered the program directly from high school (*n* = 259, 71%), those who had completed tertiary study (*n* = 87, 24%), and international students (*n* = 19, 5%). The age at course entry ranged from 17–35 years (mean 19.8, SD 2.6 years). Seven participants (2%) did not complete the course.

### Procedures

An online survey hosted via the Qualtrics™ platform comprised informed consent and four sections of measures. Age, gender (male, female, other), level and location of study completed before beginning their course were collected in section one, followed by three validated questionnaires presented in randomised order to mitigate question order bias.

### Questionnaires

#### NEO-Five-Factor Inventory (NEO-FFI-3)

The personality domains of participants were assessed via the Neuroticism, Extraversion and Openness Five Factor Inventory–3 (NEO-FFI-3 Form S—adolescent) self-report scale [40]. This abbreviated 60-item scale, developed from the original NEO Personality Inventory, is commonly used in medical education research [18]. Each of the five personality domains is represented by twelve items rated on a five-point scale from 0 (strongly disagree) to 4 (strongly agree) [40]. Domain scores were produced by summing their respective items.

#### Revised Approaches to Studying Inventory (RASI)

The Revised Approaches to Studying Inventory (RASI) is a section of the Approaches and Study Skills Inventory for Students (ASSIST) [41]. The 52 items are rated on a scale of 1 (disagree) to 5 (agree). Items represent the dimensions of deep, strategic and superficial approaches to learning, with underlying subscales. While the three categories have been verified via factor analysis in general student populations [42–44] and occupational therapy students [45], the scale's authors advise confirming the item factor structure for each study's population, as subscales may load on different factors [41]. In this study, Principal Component Analysis (PCA) with oblique rotation generated a four-component solution, explained in more detail in the analysis section.

#### Brief COPE

The Brief COPE is a condensed version of the Coping Orientation to Problems Experienced (COPE) scale initially developed by Carver, Scheier [46]. It is used broadly across psychology and health research [47, 48]. The 28-item measure encompasses 14 conceptual subscales of coping strategies. These are active coping, planning, using instrumental support, using emotional support, venting, behavioural disengagement, self-distraction, self-blame, positive reframing, humour, denial, acceptance, religion and substance use. Two items comprise each subscale, rated on a four-point scale from 1 (I don't usually do this at all) to 4 (I usually do this a lot). As a multidimensional tool, factor or principal components analysis has been applied in other studies to create higher order categories or for data reduction [47, 49]. In this study, PCA with oblique rotation derived a six-component solution.

#### Assessment of course performance

Participants' summative assessment results from three transition points were the outcome variables for this study (see Table 1). These were the end of Year 1 (T1), the completion of preclinical units (mid-Year 3, T2) and course completion (end of Year 4, T3). Written examinations comprised multiple choice and short answer questions on physiotherapy theory and practice and contribute summative weighting to preclinical units. Clinical performance was assessed via OSCEs in preclinical units and by direct observation of clinical practice during clinical placements in the final 1.5 years of the course. The Assessment of Physiotherapy Practice (APP), a workplace-based tool with established validity and reliability, was applied to assess performance on clinical placements [50, 51]. Assessment of clinical competencies across seven practice domains (professional behaviour, communication, assessment, analysis and planning, intervention, evidence-based practice and risk management) occur throughout a clinical placement (five weeks) rather than in a one-off examination. The APP contains 20 items, each rated on a five-point Likert scale of 0 (infrequently/rarely demonstrated) to 4 (demonstrates most performance indicators to an excellent standard), which were scored relative to the standard of a new graduate in clinical practice. Adding items generates a total score with a maximum of 80. The final APP scores for the first and final clinical placements were utilised for this study at T2 and T3 (see Table 1).

**Table 1:** Course assessment categories and transition points

	Course transition point			
Assessment category	T1 End Year 1	T2 Transition to clinical education Mid Year 3		T3 End of course End Year 4
Academic (knowledge)	Written examination	Written examination		
Clinical	OSCE	OSCE	CP 1 (APP score)	CP 5 (APP score)
In-Course Assessments	Yes	Yes		Yes

*OSCE* Objective structured clinical examination, *CP* Clinical placement, *APP* Assessment of physiotherapy practice

In-course assessments (e.g. assignments, presentations) that contributed to unit grades were considered separately at each transition point as markers of students' performance separate from summative examinations (i.e. written or OSCE) or clinical placement performance. Table 2 displays the relative contribution of each assessment category to the course. If repeat performance of any assessment was required due to an unsatisfactory outcome, the first attempt was included in the analysis.

**Table 2:** Weightings of assessment categories contributing to the course

Assessment category	Assessment	Contribution to course (4 years)	Range of weighting within one course year
Academic	Written Examinations	23%	15—32.5%
Clinical	OSCEs	15%	15—27.5%
	Clinical Placements	22%	37.5—50%
In-Course Assessments	Multiple	40%	32.5—50%
Total		100%	

*OSCE* Objective structured clinical examination

#### Analysis

Completed survey data were screened for missing values or potentially insincere responses by reviewing response time and invariant responses [52]. Data from 363 participants were initially included in the analysis for each questionnaire. The suitability of data from RASI and Brief COPE questionnaires for PCA was confirmed via an adequate number of variables with correlations *r* > 0.30, sufficient sampling adequacy shown via Kaiser–Meyer–Olkin values exceeding the minimum required value of 0.60, and Bartlett's test of sphericity reaching statistical significance [53].

PCA with oblique rotation (direct oblimin) was performed where a combination of Kaiser's criterion (eigenvalues), scree plots and parallel analysis [54] were consulted to assess the most suitable number of components for each questionnaire [53]. As the sample size exceeded 350, items were retained throughout this iterative process if component loadings and communalities exceeded 0.30 while not cross-loading on another component > 0.30 [55].

PCA of the RASI responses derived a four-component solution with acceptable reliability coefficients. Twenty-eight items were retained that explained 46% of the total data variance (see Table 3). Two components represented *'strategic'* and *'deep'* learning approaches described in the initial questionnaire. Whereas items from the *'surface'* learning approach separated into the components of *'fear of failure'* representing worry or anxiety related to poor assessment performance, and *'lack of purpose'* describing an approach lacking interest or direction and questioning the value of the course material.

**Table 3:** Retained items of each component of the RASI derived via principal component analysis and their component loadings

	***Components***			
	1	2	3	4
	Strategic	Deep	Fear of failure	Lack of purpose
Cronbach’s alpha coefficients for derived components	.86	.77	.76	.68
***Item Component Loadings***				
I organise my study time carefully to make the best use of it	.82			
I generally make good use of my time during the day	.78			
I typically work steadily through the semester, rather than leave it all until the last minute	.75			
I put a lot of effort into studying because I’m determined to do well	.68			
I don’t find it at all difficult to motivate myself	.66			
I’m pretty good at getting down to work whenever I need to	.65			
I think I’m quite systematic and organised when it comes to revising for assessments or exams	.63			
I manage to find conditions for studying which allow me to get on with my work easily	.61			
I usually feel that I’m getting on well, and this helps me put more effort into my work	.49			
When I read an article or book, I try to find out for myself exactly what the author means		.65		
Ideas in course books or articles often set me off on long chains of thought of my own		.62		
I sometimes get ‘hooked’ on academic topics and feel I would like to keep on studying them		.61		
I like to play around with ideas of my own even if they don’t get me very far		.59		
When I’m working on a new topic, I try to see in my own mind how all the ideas fit together		.58		
When I am reading or studying, I stop from time to time to reflect on what I am trying to learn from it		.57		
It’s important for me to be able to follow the argument, or to see the reason behind things		.55		
I look at the evidence carefully and try to reach my own conclusion about what I’m studying		.52		
I usually set out to understand for myself the meaning of what we have to learn		.51		
I try to relate ideas I came across to those in other topics or other units whenever possible		.51		
I often seem to panic if I get behind with my work			.78	
I often worry about whether I’ll be able to cope with the work in this course properly			.76	
Often I lie awake worrying about work I think I won’t be able to do			.73	
I often felt as if I was drowning in the sheer amount of material we were having to cope with			.71	
I’m not really interested in this course, but I have to take it for other reasons				.77
When I look back, I sometimes wonder why I ever decided to come here				.73
There’s not much of the work for this course that I find interesting or relevant				.65
Much of what I study makes little sense: it’s like unrelated bits and pieces				.53
Often I find myself wondering whether the work I have done here is really worthwhile				.51

PCA of Brief COPE responses generated a six-component solution with acceptable internal consistency that retained 16 items and explained 76% of total data variance (see Table 4). Four components reflected the subscale structure of the Brief COPE and were titled accordingly as *'humour'*, *'substance use'*, *'self-blame'* and *'positive-reframing'*. The remaining components were named in consultation with three expert and independent education researchers as *'support seeking'*, representing strategies to seek helpful advice and emotional support from others, and *'solution focussed',* describing strategies of constructive actions and planning. Component scores for both questionnaires were generated by summing their item scores, which were utilised for subsequent analysis. The reliability coefficients (Cronbach's alpha) of all questionnaires are shown in Table 5.

**Table 4:** Retained items of each component of the Brief COPE derived via principal component analysis and their component loadings

	***Components***					
	1	2	3	4	5	6
	Support seeking	Humour	Solution focussed	Substance use	Self-blame	Positive reframing
Cronbach’s alpha coefficients for derived components	.87	.84	.77	.91	.75	.77
***Item Component Loadings***						
I get emotional support from others	.87					
I try to get advice or help from other people about what to do	.84					
I get help and advice from other people	.84					
I get comfort and understanding from someone	.83					
I make fun of the situation		.93				
I make jokes about it		.93				
I concentrate my efforts on doing something about the situation I'm in			.78			
I try to come up with a strategy about what to do			.77			
I think hard about what steps to take			.75			
I try to take action to make the situation better			.73			
I use alcohol or drugs to help me get through it				.96		
I use alcohol or other drugs to make myself feel better				.94		
I blame myself for things that happened					.90	
I criticise myself					.86	
I look for something good in what is happening						.90
I try to see it in a different light, to make it seem more positive						.89

**Table 5:** Questionnaire components and Cronbach’s alpha coefficients

*Questionnaire*	*Component*	*No of items*	*Cronbach's alpha*
NEO-FFI-3 *n* = 363	Conscientiousness	12	.86
	Neuroticism	12	.85
	Openness to experience	12	.77
	Extraversion	12	.88
	Agreeableness	12	.81
RASI *n* = 362	Strategic	9	.86
	Deep	10	.77
	Fear of failure	4	.76
	Lack of purpose	5	.68
Brief COPE *n* = 363	Support seeking	4	.87
	Humour	2	.84
	Solution focussed	4	.77
	Substance use	2	.91
	Self-blame	2	.75
	Positive reframing	2	.77

Predictor variables for analysis included the component scores from each questionnaire and age upon course entry, gender and cohort year. Outcome variables were the results of course assessments, as outlined in Table 1.

Multivariate and univariate outliers were identified, and a range of 2–8% of participants were excluded from analysis. See Tables 6 and 7 for the final sample size per questionnaire and outcome. The minimum sample size for 80% power and α = 0.05 was confirmed by applying Tabachnick and Fidell’s [56] guidelines regarding the number of predictor variables, where a minimum sample of 146 would satisfy regression analysis with the most predictors. Pearson correlation analyses explored relationships between predictor and outcome variables. As prior tertiary study was strongly correlated with age (*r* = 0.70) it was not included as a separate predictor. Regression analysis was deemed inappropriate for the Brief COPE data due to multiple low correlations < 0.30 [53]. Analysis of variance (ANOVAs) confirmed statistically significant differences between cohort years on three outcomes; therefore, cohort year was included in regression analyses. The course completion groups were compared with Mann–Whitney analysis due to the small sample size (n = 7) and score distribution of the non-completion group.

Hierarchical multiple regression (Enter method) was conducted to examine predictive relationships between questionnaires (NEO-FFI-3 and RASI) and outcome variables. As the sample included multiple cohorts and was predominantly female (reflecting the student population), predictors were added in the following sequence: Step 1) cohort year (dichotomised), Step 2) age, gender (dichotomised male/female, as no other gender was recorded), Step 3) component scores from each questionnaire. This order allowed for the exploration of the effects of the questionnaire responses separate to those of cohort year, age and gender.

The standardised regression coefficients (*β),* adjusted *R*^2^, and Cohen's *f*^2^ are reported for each model. The effect size represented by Cohen’s *f*^2^ is interpreted as *f*^2^ ≥ 0.02 = ‘small’ *f*^2^ ≥ 0.15 = ‘moderate’ and *f*^2^ ≥ 0.35 = large. SPSS for Windows, Version 25 [57] was utilised to perform all analyses.

## Results

### Personality (NEO-FFI-3)

Significant regression models with small to moderate effect sizes of all performance outcomes, except for T2 OSCE, were found (Table 6). After controlling for the effects of cohort year, gender and age, conscientiousness was a prominent positive predictor contributing to significant models; that is, a higher conscientiousness score predicted a higher score in the outcome evaluated. To a lesser extent, higher scores on extraversion predicted Clinical Placement scores. Higher scores on openness to experience predicted lower In-Course Assessment scores at T1 and T3 and lower Clinical Placement scores at T3.

**Table 6:** Regression coefficients (β) from final regression models with personality domains measured by the NEO-FFI-3

		Written Examination		OSCE		Clinical Placement		In-Course Assessment		
		T1	T2	T1	T2	T2	T3	T1	T2	T3
	*n*	342	346	347	347	345	337	350	356	351
Cohort Year (Cohort 2 = 0)	1	.13^*^	.18^**^	-.03	.04	.00	.02	.03	.03	.01
	3	.07	-.21^***^	.16^*^	-.02	.08	.04	.01	-.04	-.05
	4	-.06	.14^*^	.02	-.02	-.02	.01	.04	-.03	-.02
	5	.25^***^	.11	.17^*^	.14^*^	.06	.04	.17^**^	.15^**^	.21^***^
	6	.21^**^	.35^***^	.17^*^	-.04	.04	-.04	.20^***^	.18^*^	.25^***^
Gender (Female = 0)		-.04	-.08	-.12^*^	-.01	-.06	-.13^*^	-.09	-.12^*^	-.17^**^
Age		.12^*^	.03	.12^*^	-.03	.04	.04	.25^***^	.23^***^	.17^**^
Personality domains	A	.04	.00	.02	.07	-.06	-.02	.04	.04	.04
	C	.19^**^	.22^***^	.22^***^	.04	.27^***^	.13^*^	.25^***^	.28^***^	.28^***^
	E	-.10	-.08	.03	.04	.12^*^	.15^**^	-.09	-.06	-.02
	N	-.04	.03	-.06	-.08	.01	.01	-.06	-.04	-.02
	O	.02	.03	.00	-.04	-.07	-.11^*^	-.13^**^	-.09	-.11^*^
	Adj *R*^2^	**.14**^Δ***^	**.30**^Δ***^	**.12**^Δ***^	.01	**.08**^Δ***^	**.05**^Δ***^	**.24**^Δ***^	**.23**^Δ***^	**.29**^Δ***^
Cohen’s *f*^2^		.16	.43	.18		.08	.05	.31	.30	.41

*NEO-FFI-3* NEO five factor inventory – 3, *OSCE* Objective structured clinical examination, *A* Agreeableness, *C* Conscientiousness, *E* Extraversion, *N* Neuroticism, *O* Openness to experience

^*^p < .05

^**^ p < .01

^***^p < .001

Δ = significant change statistic (increase in variance) at Step 3, all β coefficients are standardised

### Learning approaches (RASI)

Table 7 contains regression models with moderate to large effect sizes for In-Course Assessments and Written Examinations at each transition point. A strategic learning approach was a positive predictor within significant models after controlling for the effects of cohort year, gender and age, with the largest coefficients for T2and T3 In-Course Assessments. The significant models for clinical assessment outcomes, T1 OSCE and T2 Clinical Placement demonstrated moderate and small effect sizes, respectively. A lower score in these clinical assessments was predicted in part by a higher lack of purpose (surface learning approach). A similar relationship was also seen between the lack of purpose learning approach and lower T3 In-Course Assessment scores.

**Table 7:** Regression coefficients (β) from final regression models with approaches to learning measured by the RASI

		Written Examination		OSCE		Clinical Placement		In-Course Assessment		
		T1	T2	T1	T2	T2	T3	T1	T2	T3
	*n*	343	345	345	342	336	321	335	344	345
Cohort Year (Cohort 2 = 0)	1	.12	.16^*^	-.06	.04	-.03	.01	.02	.00	.01
	3	.07	-.22^***^	.11	-.04	.03	.00	.02	-.02	-.07
	4	-.06	.15*	.00	.00	-.05	.01	.12	-.01	.01
	5	.27^***^	.10	.12	.12	.04	.00	.23^***^	.12^*^	.20^**^
	6	.21^**^	.31^***^	.12^*^	-.05	.01	-.11	.23^***^	.17*	.23^***^
Gender (female = 0)		-.04	-.10	-.08	.01	-.07	-.06	-.10^*^	-.18^***^	-.16^*^
Age		.11^*^	.03	.11^*^	-.06	.06	-.02	.29^***^	.21^***^	.16^**^
Learning approaches Strategic		.18^**^	.20^***^	.18^**^	.03	.14^*^	.08	.19^***^	.28^***^	.28^***^
Deep		.04	.04	-.01	.00	-.10	-.04	-.08	-.06	-.10*
Fear of failure		-.10	.02	.02	-.02	-.09	.07	-.05	-.04	.03
Lack of purpose		.01	-.04	-.19^***^	-.13^*^	-.13^*^	-.15^*^	-.07	-.10	-.12^*^
	Adj *R*^2^	**.15**^Δ***^	**.29**^Δ***^	.**14**^Δ***^	.01	**.04**^Δ*^	.01	**.27**^Δ***^	**.26**^Δ***^	**.30**^Δ***^
Cohen’s *f*^2^		.18	.41	.16		.04		.37	.34	.43

*RASI* Revised approaches to studying inventory, *OSCE* Objective structured clinical examination

^*^p < .05

^**^ p < .01

^***^p < .001

Δ = significant change statistic (increase in variance) at Step 3, all β coefficients are standardised

### Coping strategies

Pearson correlational analysis revealed few statistically significant relationships between the Brief COPE components and course performance outcomes (Table 8). Support seeking strategies were weakly correlated with both Clinical Placements and T3 In-Course Assessment. Higher scores on solution focussed strategies were weakly correlated with T1 Written Examination and T2 Clinical Placement.

**Table 8:** Pearson’s correlation coefficients (r) for Brief COPE components and course performance outcomes

			Support seeking	Humour	Solution focussed	Substance use	Self-blame	Positive reframing
		*n*	*r*	*r*	*r*	*r*	*r*	*r*
Written Examination	T1	361	.08	-.05	.11^*^	.00	-.02	-.01
	T2	358	.07	-.05	.10	-.01	-.04	-.02
OSCE	T1	361	.08	.02	.09	-.04	-.05	.00
	T2	358	.02	.07	.03	.01	-.10	-.04
Clinical Placement	T2	357	.14^**^	.03	.13^*^	.00	-.05	.03
	T3	349	.11^*^	.00	.10	-.02	-.03	-.06
In-Course Assessment	T1	360	.01	-.09	.05	-.04	-.02	-.01
	T2	362	.05	-.07	.06	-.07	.01	-.02
	T3	357	.16^**^	-.05	.08	-.08	.00	.02

^*^p < .05

^**^p < 0.01

### Course completion

Mann–Whitney analysis demonstrated that non-course completers had lower median scores for coping strategies of support seeking (U(N_course complete_ = 356, N_course non-complete_ = 7) = 595.00, *z* = -2.381, *p* = 0.017) and humour (U = 600.00, *z* = -2.380, *p* = 0.017). This group also had a higher median score in a lack of purpose (surface) approach to learning (U = 760.5, *z* = -0.754, *p* = 0.043).

## Discussion

This study demonstrates several important relationships between personal characteristics of undergraduate physiotherapy students and their assessment performance and course progression. In particular, conscientiousness and a strategic approach to learning predicted better performance across written examinations, OSCEs, clinical placements and other in-course assessments. A lack of purpose (i.e., a surface) approach to learning predicted poorer performance on clinical placement and OSCE and was a distinguishing feature of those students who did not complete the course. In contrast, support seeking coping strategies were more frequently adopted by those who successfully completed the degree and were weakly associated with higher clinical placement ratings.

Undergraduate physiotherapy students in Australia typically have four years to achieve graduate competencies that allow registration for independent, unsupervised practice potentially in a primary care setting. To achieve graduate competencies, students complete demanding curricula [30] and must reach the minimum standard of a competent new graduate physiotherapist to pass clinical placements. The current study found that conscientiousness was a positive predictor of most outcomes considered beyond the effects of cohort, age and gender. As conscientiousness represents facets of organisation, self-discipline, and striving for achievement [40] it is therefore not a surprising predictor of assessment performance in this context and is consistent with Lievens et al.’s [4] longitudinal study with medical students. In the current study, conscientiousness was a weaker predictor of the final clinical placement score in comparison to final in-course assessments, whereas Lievens et al. reported conscientiousness gained strength as a predictor in the final year [4]. Although specific clinical performance measures were not reported as the outcome examined was yearly grade point averages [4]. We also found that higher scores in extraversion and lower scores in openness to experience were weaker predictors of clinical placement scores. Expression of warmth and being socially skilled (i.e., features of extraversion) appear to be relevant to performance in clinical settings where communication, interpersonal skills and assertiveness are of benefit [4, 8]. However, in contrast with Lievens et al*.* [4], this study found relationships between being less open to new ideas and experiences and clinical performance and in-course assessment in the later years of the course. Those who score lower on the openness personality domain prefer familiarity over novel situations and tend to behave in a conventional and conservative manner [40]. As students gain experience in clinical settings, these findings may indicate an increasing familiarity and confidence with the clinical environment and the expectations of clinical and other in-course assessments. Depending on the clinical setting, conventional or cautious behaviour could also be perceived (and rated) positively by educators. However, Milne et al. [11] reported that a steady and conscientious behavioural style differentiated physiotherapy students who received lower scores on the APP. The authors argued that this style could present as quiet or withdrawn when under pressure which may be construed as lacking knowledge or competence [11]. In our study, the contribution of low openness is weak. Future research could explore this particular domain in more detail, potentially alongside the impacts of the clinical stream or setting or specific domains of the APP.

In this study, a strategic approach to learning was a predictor of most outcomes. As with conscientiousness, this finding may reflect the demanding nature of the curriculum physiotherapy students undertake. While we did not specifically examine interactions between the personal characteristics measured, relationships between conscientiousness and strategic or deep learning approaches have been previously reported. Swanberg and Martinsen [22] demonstrated that a strategic approach to learning mediated the effect of conscientiousness on academic performance in psychology students and accounted for unique variance beyond conscientiousness. Another notable finding of the current study is that a lack of purpose (surface) learning approach predicted lower clinical performance scores (measured on OSCEs and clinical placements). Poorer performance on academic and clinical outcomes by students reporting surface approaches is consistent with recent longitudinal studies in medical students [6, 58]. The disorganisation of the surface learning approach may increase the difficulty of integrating previously learned content, intensifying the challenge of performing in clinical settings or drawing information together from across the course. These learners may also be less aware of how to apply preclinical content to clinical practice [59].

A lack of purpose (surface) approach to learning was also featured in the subgroup who did not complete the course. This group also demonstrated lower scores on support seeking and humour coping strategies. Additionally, support seeking coping strategies had a weak relationship with clinical placement scores. Given the challenges of clinical education, from the students’ perspective, we hypothesised that adaptive coping strategies may link to performance in a clinical environment. Lower support seeking scores may reflect a reduced awareness of the need for support or a lack of access to social support resulting in stress, poorer performance [8] and course withdrawal. Screening students in the early years of the course for surface learning approaches or lower tendencies in support seeking coping strategies may assist in identifying individuals at risk of poor performance or course withdrawal, particularly when transitioning to clinical placements. Such information could assist educators in monitoring these students and offering tailored support.

Although motivation was not specifically assessed, personal characteristics considered in this study that are related to improved performance during physiotherapy training and successful course completion likely reflect a learner who presents as motivated. A learner who is conscientious, strategic, and seeks support or looks for solutions when under stress is likely one who is motivated to achieve in the learning environment and potentially also in the profession. While a broad motivation to practice in the profession can be considered during course selection, and was for the participants in this study, exploring learning-specific motivation may also be valuable. Particular consideration could also be given to pedagogical approaches that stimulate interest, foster problem-solving and reflections on course content, and assesses knowledge application rather than pure recall to facilitate the application of deeper-oriented learning approaches [59–61]. Although individual students’ perceptions of learning contexts are prominent influences of their learning approaches, curriculum and cognitive overload that impede the above processes are more likely to contribute to students applying a surface approach to their learning [60, 62]. There is an increasing need for physiotherapists in the population, with specific demand in Australian aged care and rural settings particularly informing this study [63–65]. However, the expensive and intensive nature of health professional training [1], the significant costs associated with failing [66], and the fact that students who withdraw during the course cannot be replaced add to the challenges of ensuring a sufficient workforce. Therefore, identifying and supporting at-risk students may be facilitated by assessing learning approaches and coping strategies, especially during the challenging transition to clinical education as occurs in the Australian undergraduate context.

Limitations to this study are that it is observational and from a single institution, so the findings may not generalise to other contexts. However, the results are consistent with reports from other health professional student cohorts. Also, while not unusual in a study of this nature, the relationships reported are relatively weak. This study contributes to a relatively unexplored area in undergraduate physiotherapy education, investigating the relationships of students’ personal characteristics to academic and clinical summative assessment outcomes. Further research could build on these findings with prospective study designs exploring the interactions between personal and behavioural characteristics and their relationships to course outcomes, attrition, patient outcomes or career satisfaction and longevity.

## Conclusions

There is unlikely to be a dominant characteristic that is the 'silver bullet' for predicting achievement in health professional training or careers. More likely, a range of factors and their inter-relationships will contribute to meaningful professional practice outcomes, as outlined in expected graduate competencies. The results of this study emphasise the importance of a conscientious and a strategic learning approach during physiotherapy training to influence both academic and clinical performance. Identifying learners with a surface approach to learning and lower tendencies of support seeking coping strategies early in their training could assist educators in supporting those deemed at risk of non-completion. Given the substantial expense of health professions training and the necessity to produce competent clinicians who will persist in their careers to develop expertise, these findings may be relevant to physiotherapy educators to enhance students’ training experience and minimise attrition.

## Data Availability

The datasets analysed during the current study are not publicly available for privacy reasons but are available from the corresponding author upon reasonable request.

## Abbreviations

GPA: Grade Point Average
OSCE: Objective Structured Clinical Examination
NEO-FFI-3: Neuroticism, Extraversion and Openness Five Factor Inventory–3
RASI: Revised Approaches to Studying Inventory
ASSIST: Approaches and Study Skills Inventory for Students
PCA: Principal component analysis
Brief COPE: Brief Coping Orientation to Problems Experienced

## References

[1] Segal L, Marsh C, Heyes R (2017). The real cost of training health professionals in Australia it costs as much to build a dietician workforce as a dental workforce. J Health Serv Res Policy.

[2] Physiotherapy Board of Australia, Physiotherapy Board of New Zealand. Physiotherapy practice thresholds in Australia and Aotearoa New Zealand. 2015.[03 March 2020]. Available from: https://physiocouncil.com.au/media/1020/physiotherapy-board-physiotherapy-practice-thresholds-in-australia-and-aotearoa-new-zealand-6.pdf

[3] Chisholm-Burns MA, Berg-Poppe P, Spivey CA, Karges-Brown J, Pithan A (2021). Systematic review of noncognitive factors influence on health professions students’ academic performance. Adv Health Sci Educ.

[4] Lievens F, Ones DS, Dilchert S (2009). Personality scale validities increase throughout medical school. J Appl Psychol.

[5] McLaughlin K, Moutray M, Muldoon OT (2008). The role of personality and self-efficacy in the selection and retention of successful nursing students: a longitudinal study. J Adv Nurs.

[6] Piumatti G, Abbiati M, Gerbase MW, Baroffio A (2021). Patterns of Change in Approaches to Learning and Their Impact on Academic Performance Among Medical Students: Longitudinal Analysis. Teach Learn Med.

[7] Richardson M, Abraham C, Bond R (2012). Psychological correlates of university students' academic performance: A systematic review and meta-analysis. Psychol Bull.

[8] Haight SJ, Chibnall JT, Schindler DL, Slavin SJ (2012). Associations of medical student personality and health/wellness characteristics with their medical school performance across the curriculum. Acad Med.

[9] Mercer A, Hay M, Hodgson WC, Canny BJ, Puddey IB (2018). The relative predictive value of undergraduate versus graduate selection tools in two Australian medical schools. Med Teach.

[10] Paynter S, Iles R, Hay M (2021). An investigation of the predictive validity of selection tools on performance in physiotherapy training in Australia. Physiotherapy.

[11] Milne N, Louwen C, Reidlinger D, Bishop J, Dalton M, Crane L (2019). Physiotherapy students’ DiSC behaviour styles can be used to predict the likelihood of success in clinical placements. BMC Med Educ.

[12] Van Hoek G, Portzky M, Franck E (2019). The influence of socio-demographic factors, resilience and stress reducing activities on academic outcomes of undergraduate nursing students: A cross-sectional research study. Nurse Educ Today.

[13] McCrae, RR, Costa Jr, PT. The five-factor theory of personality. In: John OP, Robins RW, Pervin LA editors. Handbook of personality: Theory and research, 3rd ed. New York: The Guilford Press; 2008. p. 159–81.

[14] Chamorro-Premuzic T, Furnham A (2008). Personality, intelligence and approaches to learning as predictors of academic performance. Personality Individ Differ.

[15] Ferguson E, James D, O'Hehir F, Sanders A, McManus IC (2003). Pilot study of the roles of personality, references, and personal statements in relation to performance over the five years of a medical degree. BMJ.

[16] Schrempft S, Piumatti G, Gerbase MW, Baroffio A (2021). Pathways to performance in undergraduate medical students: role of conscientiousness and the perceived educational environment. Adv Health Sci Educ.

[17] Ferguson E, Semper H, Yates J, Fitzgerald JE, Skatova A, James D (2014). The ‘Dark Side’ and ‘Bright Side’ of Personality: When Too Much Conscientiousness and Too Little Anxiety Are Detrimental with Respect to the Acquisition of Medical Knowledge and Skill. PLoS ONE.

[18] Hojat M, Erdmann JB, Gonnella JS (2013). Personality assessments and outcomes in medical education and the practice of medicine: AMEE Guide No. 79. Med Teacher.

[19] Buining EM, Kooijman MK, Swinkels ICS, Pisters MF, Veenhof C (2015). Exploring physiotherapists’ personality traits that may influence treatment outcome in patients with chronic diseases: a cohort study. BMC Health Serv Res.

[20] Diseth Å (2003). Personality and approaches to learning as predictors of academic achievement. Eur J Pers.

[21] Poropat AE, Corno L, Anderman EM (2015). Beyond the shadow: The role of personality and temperament in learning. Handbook of educational psychology.

[22] Swanberg AB, Martinsen ØL (2010). Personality, approaches to learning and achievement. Educ Psychol.

[23] Entwistle NJ, Ramsden P (1983). Understanding Student Learning.

[24] Bonsaksen T, Magne TA, Stigen L, Gramstad A, Åsli L, Mørk G (2021). Associations between occupational therapy students' academic performance and their study approaches and perceptions of the learning environment. BMC Med Educ.

[25] Feeley A-M, Biggerstaff DL (2015). Exam Success at Undergraduate and Graduate-Entry Medical Schools: Is Learning Style or Learning Approach More Important? A Critical Review Exploring Links Between Academic Success, Learning Styles, and Learning Approaches Among School-Leaver Entry (“Traditional”) and Graduate-Entry (“Nontraditional”) Medical Students. Teach Learn Med.

[26] Piumatti G, Abbiati M, Baroffio A, Gerbase MW (2019). Associations between motivational factors for studying medicine, learning approaches and empathy among medical school candidates. Adv Health Sci Educ.

[27] McManus IC, Keeling A, Paice E (2004). Stress, burnout and doctors' attitudes to work are determined by personality and learning style: A twelve year longitudinal study of UK graduates. BMC Medicine.

[28] DaLomba E, Mansur S, Bonsaksen T, Greer MJ (2021). Exploring graduate occupational and physical therapy students’ approaches to studying, self-efficacy, and positive mental health. BMC Med Educ.

[29] Hayes KW, Sanders B, Healey WE (2010). Students' Study Approaches in a New Curriculum. J Phys Ther Educ.

[30] Walsh JM, Feeney C, Hussey J, Donnellan C (2010). Sources of stress and psychological morbidity among undergraduate physiotherapy students. Physiotherapy.

[31] Delany C, Miller KJ, El-Ansary D, Remedios L, Hosseini A, McLeod S (2015). Replacing stressful challenges with positive coping strategies: a resilience program for clinical placement learning. Adv Health Sci Educ.

[32] Connor-Smith J, Flachsbart C (2007). Relations Between Personality and Coping: A Meta-Analysis. J Pers Soc Psychol.

[33] de la Fuente, J, Fernández-Cabezas, M, Cambil, M, Vera, MM, González-Torres, MC, Artuch-Garde, R. Linear Relationship between Resilience, Learning Approaches, and Coping Strategies to Predict Achievement in Undergraduate Students. Frontiers in Psychology. 2017;8:1039. 10.3389/fpsyg.2017.01039. 10.3389/fpsyg.2017.01039 PMC5492468 28713298

[34] Skinner EA, Edge K, Altman J, Sherwood H (2003). Searching for the structure of coping: A review and critique of category systems for classifying ways of coping. Psychol Bull.

[35] Park CL, Adler NE (2003). Coping Style as a Predictor of Health and Well-Being Across the First Year of Medical School. Health Psychol.

[36] Williams P, Mueller K, Carroll H, Cornwall M, Denney L, Jroneberger L (2018). Patterns of Academic Burnout, Emotional Distress, and Coping in Physical Therapy Students. Int J Health Wellness Soc.

[37] Schiller JH, Stansfield RB, Belmonte DC, Purkiss JA, Reddy RM, House JB (2018). Medical Students' Use of Different Coping Strategies and Relationship With Academic Performance in Preclinical and Clinical Years. Teach Learn Med.

[38] Alimoglu MK, Gurpinar E, Mamakli S, Aktekin M (2011). Ways of coping as predictors of satisfaction with curriculum and academic success in medical school. Adv Physiol Educ.

[39] Tucker B, Jones S, Mandy A, Gupta R (2006). Physiotherapy students' sources of stress, perceived course difficulty, and paid employment: Comparison between Western Australia and United Kingdom. Physiother Theory Pract.

[40] McCrae, RR, Costa Jr, PT. NEO inventories for the NEO Personality Inventory-3 (NEO-PI-3), NEO Five-Factor Inventory-3 (NEO-FFI-3), NEO Personality Inventory-Revised (NEO PI-R): Professional manual. Lutz: PAR; 2010.

[41] Entwistle, NJ, McCune, V, Tait, H. Approaches and Study Skills Inventory for Students (ASSIST) (incorporating the Revised Approaches to Studying Inventory (RASI). Report of the development and use of the inventories. 2013. Available from: https://www.researchgate.net/publication/50390092_Approaches_to_learning_and_studying_inventory_ASSIST_3rd_edition

[42] Byrne M, Flood B, Willis P (2004). Validation of the approaches and study skills inventory for students (ASSIST) using accounting students in the USA and Ireland: a research note. Acc Educ.

[43] Entwistle, NJ, Tait, H, McCune, V. Patterns of response to an approaches to studying inventory across contrasting groups and contexts. European Journal of Psychology of Education. 2000;15:33–48.

[44] Speth CA, Namuth DM, Lee DJ (2007). Using the ASSIST short form for evaluating an information technology application: validity and reliability issues. Informing Sci J.

[45] Bonsaksen T, Småstuen MC, Thørrisen MM, Fong K, Lim HB, Brown T (2019). Factor analysis of the Approaches and Study Skills Inventory for Students in a cross-cultural occupational therapy undergraduate student sample. Aust Occup Ther J.

[46] Carver CS, Scheier M, Weintraub J (1989). Assessing coping strategies: A theoretically based approach. J Person Social Psych.

[47] Hagan T, Fishbein JN, Nipp R, Jacobs J, Traeger L, Irwin K (2017). Coping in Patients With Incurable Lung and Gastrointestinal Cancers: A Validation Study of the Brief COPE. J Pain Symptom Manag.

[48] Simmons, CA, Lehmann, P. Tools for strengths-based assessment and evaluation: Springer Publishing Company; 2012.

[49] Baumstarck K, Alessandrini M, Hamidou Z, Auquier P, Leroy T, Boyer L (2017). Assessment of coping: a new french four-factor structure of the brief COPE inventory. Health Qual Life Outcomes.

[50] Dalton M, Davidson M, Keating J (2011). The Assessment of Physiotherapy Practice (APP) is a valid measure of professional competence of physiotherapy students: a cross-sectional study with Rasch analysis. J Physiother.

[51] Dalton M, Davidson M, Keating JL (2012). The Assessment of Physiotherapy Practice (APP) is a reliable measure of professional competence of physiotherapy students: a reliability study. J Physiother.

[52] DeSimone JA, Harms PD, DeSimone AJ (2015). Best practice for data screening. J Org Behaviour.

[53] Pallant, J. SPSS Survival Manual. 6th ed: Allen & Unwin; 2016.

[54] Watkins, MW. Monte Carlo PCA for Parallel Analysis (Computer Software). State College: Ed & Psych Associates; 2000.

[55] Hair, JF, Black, WC, Babin, BJ, Anderson, RE. Multivariate data analysis. 7th ed. Essex: Pearson Education Limited; 2014.

[56] Tabachnick, BG, Fidell, LS. Using Multivariate Statistics: Pearson New International Edition. 6th ed. Essex (England): Pearson Education Limited; 2013.

[57] IBM Corp. IBM SPSS Statistics for Windows, Version 25.0. Armonk: IBM Corp; 2017.

[58] Piumatti G, Guttormsen S, Zurbuchen B, Abbiati M, Gerbase MW, Baroffio A (2021). Trajectories of learning approaches during a full medical curriculum: impact on clinical learning outcomes. BMC Med Educ.

[59] Mørk G, Magne TA, Carstensen T, Stigen L, Åsli LA, Gramstad A (2020). Associations between learning environment variables and students' approaches to studying: a cross-sectional study. BMC Med Educ.

[60] Sellheim DO (2003). Educational Factors Influencing Physical Therapist Students' Approaches to Learning. J Phys Ther Educ.

[61] Wilson K, Fowler J (2005). Assessing the impact of learning environments on students' approaches to learning: comparing conventional and action learning designs. Assess Eval High Educ.

[62] Smarandache IG, Maricutoiu LP, Ilie MD, Iancu DE, Mladenovici V (2022). Students’ approach to learning: evidence regarding the importance of the interest-to-effort ratio. High Educ Res Dev.

[63] National Skills Commission (Australian Government). Skills Priority List. 2021. Available from: https://www.nationalskillscommission.gov.au/sites/default/files/2021-06/Skills%20Priority%20List%20Occupation%20List_0.pdf. [20 Dec 2021].

[64] Long J (2019). European region of the WCPT statement on physiotherapy in primary care. Prim Health Care Res Dev.

[65] Landry MD, Hack LM, Coulson E, Freburger J, Johnson MP, Katz R (2016). Workforce Projections 2010–2020: Annual Supply and Demand Forecasting Models for Physical Therapists Across the United States. Phys Ther.

[66] Foo J, Rivers G, Ilic D, Evans DJR, Walsh K, Haines T (2017). The economic cost of failure in clinical education: a multi-perspective analysis. Med Educ.

